# A pupil size, eye-tracking and neuropsychological dataset from ADHD children during a cognitive task

**DOI:** 10.1038/s41597-019-0037-2

**Published:** 2019-04-11

**Authors:** Daniel Rojas-Líbano, Gabriel Wainstein, Ximena Carrasco, Francisco Aboitiz, Nicolás Crossley, Tomás Ossandón

**Affiliations:** 1 Laboratorio de Neurociencia Cognitiva y Social, Facultad de Psicología, Universidad Diego Portales, Santiago, Chile; 2 Neurodynamics of Cognition Laboratory, Departamento de Psiquiatría, Escuela de Medicina, Pontificia Universidad Católica de Chile, Santiago, Chile; 3 Department of Psychosis Studies, Institute of Psychiatry, Psychology and Neuroscience, King’s College London, London, UK; 4 Servicio de Neurología y Psiquiatría, Hospital de Niños Dr. Luis Calvo Mackenna, Facultad de Medicina, Universidad de Chile, Santiago, Chile

**Keywords:** ADHD, Attention, Diagnostic markers

## Abstract

Attention Deficit/Hyperactive Disorder (ADHD) is diagnosed based on observed behavioral outcomes alone. Given that some brain attentional networks involve circuits that control the eye pupil, we monitored pupil size in ADHD- diagnosed children and also in control children during a visuospatial working memory task. We present here the full dataset, consisting of pupil size time series for each trial and subject. There are data from, 22 control, and 28 ADHD-diagnosed children. There are also data from a subset of 17 ADHD children that performed the task twice, on- and off-medication. In addition, our dataset also includes gaze position data from each trial and subject, and also scores from the Weschler Intelligence Scale for Children. In this context, the dataset can serve as a resource to analyze dynamic eye movement and pupil changes as a function of known behavioral changes and scores in neuropsychological tests, which reflect neurocognitive processing.

## Abstract (summary)

**Table:** 

Design Type(s)	repeated measure design • parallel group design • disease detection/diagnosis objective
Measurement Type(s)	pupil size trait
Technology Type(s)	eye tracking device
Factor Type(s)	experimental condition • age • biological sex • Medication
Sample Characteristic(s)	Homo sapiens • eye

Machine-accessible metadata file describing the reported data (ISA-Tab format)

## Background & Summary

Attention Deficit/Hyperactivity Disorder (ADHD) is defined as the presence of a set of behavioral symptoms and diagnosed accordingly. These purely behavioral criteria allow the physician to diagnose the condition based mainly on a verbal report of the observed child’s behavior. Even when it is considered to be a neurodevelopmental disorder^1–3^, and hence assumed as having a biological origin, there are no biological tests that could contribute to making the diagnosis more objective. As with other psychiatric conditions, this reflects the fact that we currently lack a causal understanding of the origins and the biological mechanisms underlying the set of symptoms. Relying exclusively on described behavioral outcomes carries the potential risk of under or over-diagnosis, which in turn implies giving pharmacologic treatment to children that might not need it, or refusing it to those that would benefit from it.

The limitations of the current diagnostic criteria have been discussed extensively elsewhere^4,5^. Some of these shortcomings could be alleviated through translational research grounded in an evidence-based approach^6,7^. Therefore, there is a need for carefully collected biological and behavioral data that could allow researchers to investigate the mechanisms underlying the described conditions.

We present here a dataset containing both behavioral and pupil size data from children performing a visuospatial working memory task. Our data include two groups, ADHD-diagnosed children, and non-diagnosed controls. It also features two sessions, off- and on-medication, from a subset of the ADHD-diagnosed children. Neuroscientific results from the experiment have been peer-reviewed and published elsewhere^8^. In that study, we found that pupil size tracks attentional processes, co-varying consistently with the relevant trial’s events (such as array image presentations) and with behavioral variables (such as performance and reaction time). We also found some differences between ADHD and control groups, albeit with important intra-group variation.

In addition to the published pupil data, the dataset we are presenting also includes gaze position data from each trial and subject, and also twelve sub-test scores from the Weschler Intelligence Scale for Children (WISC), for each participant. The raw pupil data also includes the eye blinks occurrences from each subject, which can also be analyzed as a behavioral index of dopaminergic activity.

Current neuroscientific theories of attentional processes have implicated different brain networks, whose concerted action would underlie actions such as engage in and disengage from a task, allocate and deploy attentional resources, and promote the alertness necessary to pay attention to a specific stimulus. One of these networks is the brain norepinephrine (NE) system, a diffuse collection of fibers that originates in the locus coeruleus (LC) and that projects through several brain areas, including extensive portions of the neocortex, hippocampus, midbrain and thalamus^9,10^. The LC-NE system is also involved in the control of pupil size, and several lines of evidence have shown that pupil dynamics can reflect the brain states relevant to cognition^11–15^. Pupil size, and in particular changes in pupil size during a relevant cognitive task, can be thought of as a window into the ongoing neural mechanisms underlying cognitive processes. The present dataset, which we have made publicly available^16^, contains the relevant stimuli events and behavioral events concurrent with pupil time series and eye-position data in the context of a known cognitive task designed to assess visuo-spatial attentional capabilities. In addition, we present detailed standardized neuropsychological data from each participant, which can be used to parse or cluster subjects and investigate potential relationships with eye data and task performance. Therefore, the data allows for further explorations of the link between physiology and different estimates of cognition, as well as for exploratory hypothesis testing regarding this link.

## Methods

We present in this section an expanded version of method descriptions presented in our previously published article^8^.

### Subjects and study design

A group of 50 subjects participated in this study. 28 subjects were patients diagnosed with ADHD (4 girls, age: 10.71 ± 0.54 years-old), drawn from a pool of diagnosed patients, and 22 healthy control children (4 girls, age: 11.58 ± 0.50 years-old) recruited from local schools. All ADHD children were being treated with methylphenidate at the time of the study and a subgroup of 17 ADHD patients (3 girls, age: 11.19 ± 0.86 years-old) performed the task twice, on- and off-medication (time between sessions: 180.23 ± 125.17 days). For off-medication sessions, children discontinued their medication 24 h prior to the day of testing. All ADHD subjects were diagnosed following standard procedures by a neurologist (for a complete description of diagnoses and additional cognitive and clinical assessments, see^8^). Parents consented to their children’s participation, and children signed an informed assent form. All procedures were approved by the Ethics Committee of the School of Medicine of Pontificia Universidad Católica de Chile (Protocol number 11082).

**Fig. 1:**
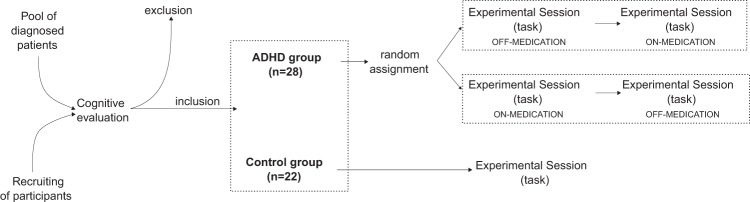
Sequence of events from subjects’ recruiting to recording data in experimental sessions. We recruited subjects from diagnosed ADHD patients and from voluntary subjects. We then performed a cognitive evaluation of subjects and selected only subjects which matched our inclusion criteria. Through this procedure we obtained our experimental groups, ADHD (n = 28 children) and Control (n = 22 children). Control children then scheduled a day to perform the task in one experimental session. ADHD patients were randomly assigned to one out of two groups, depending on the sequence of sessions (on-medication first, and then off-medication, or off-medication first, and then on-medication). On- and off-medication sessions for ADHD took place in separate days.

Subjects in the ADHD group were randomly assigned to one of two groups, where the sessions’ order (on- and off-medication) were swapped (see Fig. 1).

### Task

**Fig. 2:**
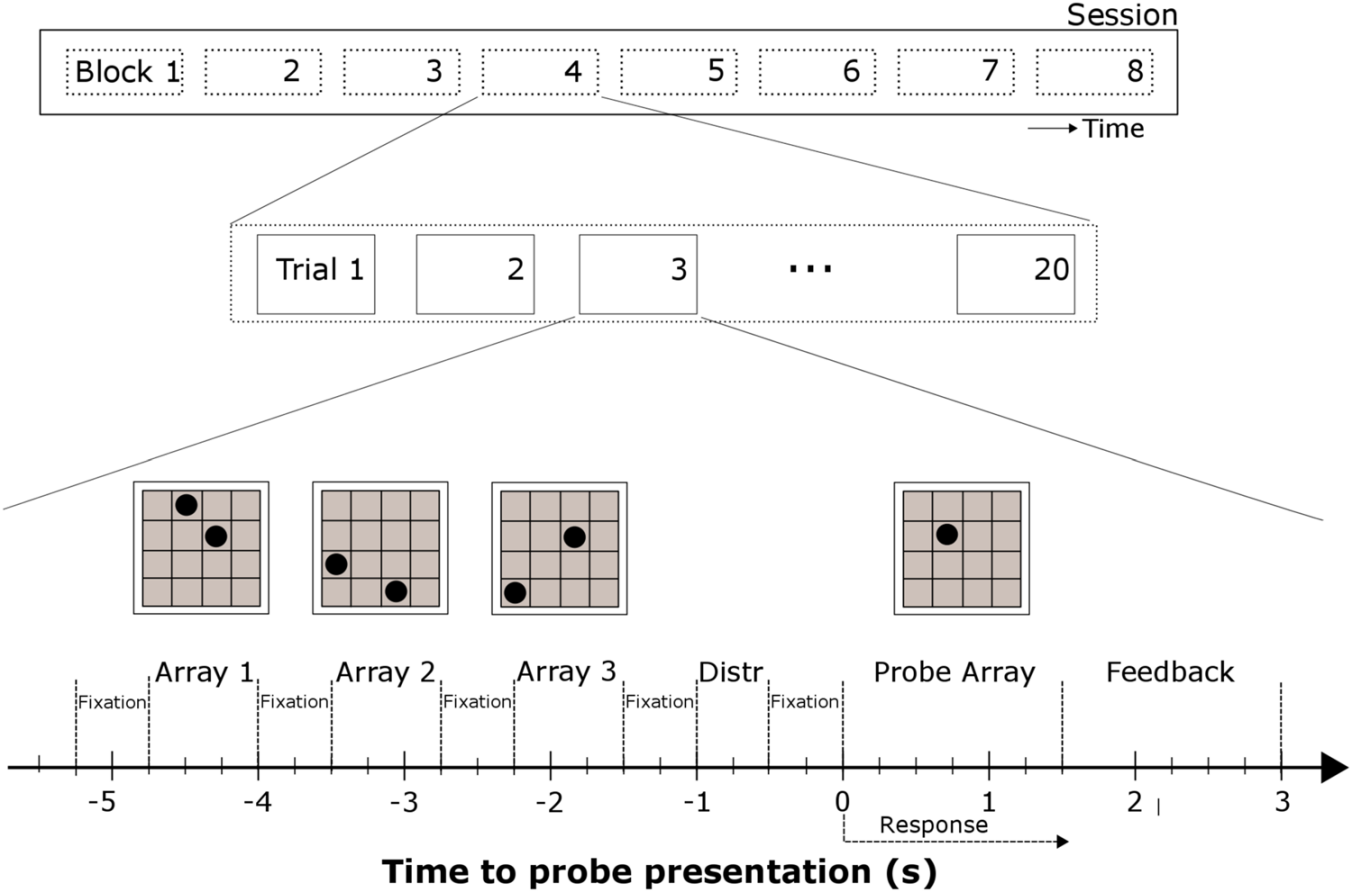
Temporal organization of an experimental session. A session consisted on eight consecutive blocks, which in turn consisted of a sequence of twenty trials. On each trial, subjects watched a sequence of three dot-arrays on a 4 × 4 grid, separated by a fixation screen with a center cross. Then a distractor (Distr) image was presented, which could be a dot array, an emotional image, a neutral image or a flat-color screen. Then a Probe dot-array was presented and the subject had to indicate if the dot in the probe had been presented in one of the previous three arrays. After 1.5 seconds, feedback was provided to the subject regarding its performance on the current trial. A similar version of this figure was previously published in ref.^8^.

Subjects performed a Sternberg-type delayed visuospatial WM task, adapted from Dolcos & McCarthy^17^. The memoranda were 1- or 2-dot arrays, with the dots located in any of the sixteen spaces of a 4 × 4 grid (See Fig. 2). On each trial, subjects were instructed to start by fixating on a black cross located at the center of the screen. After 500 ms, dot array presentation commenced. Three different dot arrays were presented on each trial. Each array was presented during 750 ms, with a 500 ms delay between arrays, during which a fixation cross was presented (see Fig. 2). After the last delay period, a distractor image was presented for 500 ms. After the distractor, a ‘probe’ dot was presented for 1.5 seconds. This was a dot within the grid, and subjects had to answer ‘yes’ if the probe dot had been presented in one of the trial’s previous arrays, or ‘no’ if it had not.

We used three distractors: (1) A task-related dot array; (2) a neutral natural image; and (3) an emotional image. In 25% of the trials, no distractor was presented. Trial types were presented randomly. There were also two trial types, according to cognitive load: In low-load trials, only one dot was presented on each image, whereas in high-load trials, two dots were presented on each image. Therefore, in low-load trials subjects had to retain the location of three dots (one per image) and in high-load trials they had to retain the location of six dots (two per image).

A total of 160 trials were presented on each session, separated in 8 blocks of 20 trials. Sessions usually lasted 30 minutes.

### Data acquisition

Pupil diameter data was acquired with Eyelink 1000 (SR Research Ltd., Mississauga, Ontario, Canada), with a 1 kHz sampling frequency. Subjects sat in front of a table containing the computer screen for image presentation and the eye tracker device. During the task, the subjects kept their head in a forehead/chin rest (SR Research Ltd.). Subjects were placed at a viewing distance of 60 cm from the computer screen.

The computer screen had a size in pixels of 1920 × 1080. Grids were squares, positioned with their center at the center of the screen. Each grid size was 200 × 200 pixels, and each division of the grid was a square of 50 × 50 pixels. The dots memoranda were a circle of 30 pixels in diameter placed in the center of the corresponding grid square.

### Neuropsychological data

All participants were tested individually on the WISC-III or the WISC-R (3 children) by trained neurologists or neuropsychologists from the clinical team at Universidad Católica de Chile. Index scores were calculated as follow: When a child’s performance on a subtest is compared to the normative sample, subtest scores are converted into scaled scores that serve as universal metrics. A scaled score of 10 is the mean and scaled scores that deviate 3 units reflect a standard deviation. After, similar subtests are combined into Primary Index Scales that have a mean of 100 and standard deviation of 15. These numbers are used to determine the performance. Classification of performance for scaled index scores are as follows: Below Average – scaled score 1 to 5; Low Average – scaled score 6 to 7; Average – scaled score 8 to 11; High Average scaled score 12 to 13; Superior – 14 to 15; Very Superior – 16 to 20. Consequently, descriptors of performance for standard WISC score ranges are as follows: Below Average – standard score below 79; Low Average – standard score 80 to 89; Average – 90 to 109; High Average – 110 to 119; Superior – 120 to 129; Very Superior – above 130.

## Data Records

**Fig. 3:**
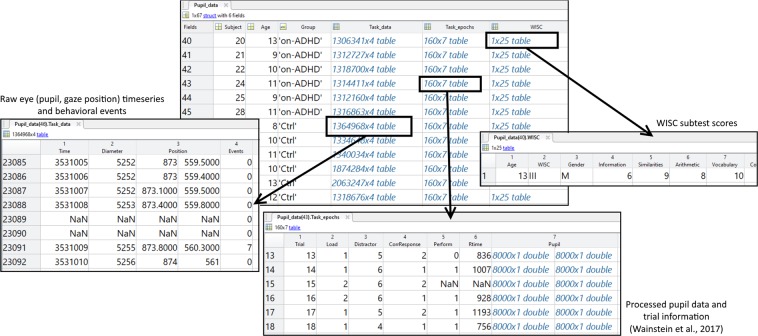
Screenshot samples of data structure ‘Pupil_data’ within the Matlab® environment. The file contains a structure array with six fields (‘Subject’, ‘Age’, ‘Group’, ‘Task_data’, ‘Task_epochs’, and ‘WISC’) and one level per session recorded. See text for details.

Data are organized in a Matlab® structure array called ‘Pupil_data’, which contains one index per session recorded (see Fig. 3). Data are provided at figshare website^16^. Given that in our design a subset of ADHD children performed the task twice (on- and off-medication), in different days, we keep these repetitions as separate sessions. The structure contains five fields, within which we located both the actual data and session information. In what follows, we describe each field in detail.

### Subject

This field codes the participant’s identity, assigning an integer to each participant.

### Age

Participant age (in years), at the moment of performing the task.

### Group

This field indicates the experimental group to which the participant was assigned to in a given session. They are labeled ‘off-ADHD’, ‘on-ADHD’ and ‘Ctrl’, indicating off-medication ADHD participant, on-medication ADHD participant, and control participant, respectively.

### Task_data

This field contains a table array in which we provide all the raw data from that participant and session. The data are arranged in four columns, with one row per timestamp. The first column, ‘Time’, provides the timestamps of the session, in milliseconds. These timestamps come from the Eyelink recording. The second column, ‘Diameter’, contains the pupil diameter, in arbitrary units, also coming from the Eyelink equipment. The third column, ‘Position’, is organized in two sub-columns, which contain the x- and y-coordinates of the gaze positions, in pixels, relative to the screen size in pixels (1920 × 1080). The fourth column, ‘Events’, contain the relevant task and behavioral events. The data in this column contain a zero if no event occurred and an integer from 1 to 10 if an event occurred at a given timestamp. These numbers then provide the event occurrence, as described. 1: Presentation of one-dot memoranda; 2: Presentation of two-dot memoranda; 3: Presentation of neutral distractor; 4: Presentation of empty- grid distractor; 5: Presentation of task-related distractor; 6: Presentation of emotional-image distractor; 7: Presentation of probe array; 8: Presentation of feedback screen; 9: Occurrence of button press with participant answering ‘yes’; 10: Occurrence of button press with participant answering ‘no’.

### Task_epoch

This field consists of a table array containing processed (filtered, interpolated) pupil data, with one epoch per trial. Rows correspond to trials and columns to different events and data. ‘Trial’ column simply indexes the trial. ‘Load’ codes the memoranda types, indicating one-dot (1) or two-dot (2), which are operationalizations of cognitive load. ‘Distractor’ codes the distractor types: neutral (3), empty- grid (4), task-related (5) and emotional-image (6). ‘CorrResponse’ contains the information about the correct response the participant was expected to give, with 1 indicating that the probe dot was indeed present in previous dot arrays and 2 indicating that the probe dot was not present. ‘Perform’ corresponds to the participant’s performance in assessing the dot probe, which could be either correct (1) or incorrect (0). The column ‘RTime’ contains the reaction time in milliseconds, which is the time elapsed between probe array presentation and the participant’s button press. If the participant did not respond in a given trial, the entry contains ‘NaN’ (not-a-number) Finally, the ‘Pupil’ column contains two vectors of size 8000 (i.e., corresponding to the 8 seconds of each trial), with the first containing the session timestamps and the second containing the pupil in z-score units. The data in *Task_epoch* corresponds to the data used in Wainstein *et al*. (2017).

### WISC

This field consists of a table array with the WISC data. Each column corresponds to a subtest within WISC and the entries contain the associated scores. Given that the test was administered only once per participant, for the same ADHD participant in on- and off-medication sessions, WISC data is simply repeated.

## Technical Validation

A total of 50 children participated in the study. 28 children were ADHD-diagnosed patients (23 boys, 5 girls, age: 10.71 ± 0.54 years old). During the duration of the study, all ADHD children were being treated with methylphenidate. For the control group, we collected data from 22 non-ADHD-diagnosed children (18 boys, 4 girls, age: 11.58 ± 0.50 years-old).

A subset of 17 ADHD-diagnosed children (14 boys, 3 girls, age: 11.19 ± 0.86 years-old) performed the task twice, on- and off- medication, in two separate sessions. Since this within-subject design could potentially be affected by learning effects, we assigned children randomly to a session order. The information indicating this order is provided in Table 1.

**Table 1:** Medication (On- or off-) sequence for the subset of ADHD-diagnosed participants who performed the task twice.

Subject ID	Session 1	Session 2
1	On	Off
2	Off	On
4	Off	On
5	On	Off
9	Off	On
11	Off	On
12	On	Off
13	On	Off
16	Off	On
17	On	Off
18	On	Off
20	On	Off
21	Off	On
22	Off	On
24	On	Off
25	On	Off
28	On	Off

Before each trial block, we applied the standard Eyelink calibration protocol to track the positions of the pupil and the corneal reflection of the right eye. A black circle (‘target’) was presented in the screen sequentially at 9 different locations (4 corners, 4 middle points of screen edge and 1 at the center) and the subject was instructed to fixate on them as they appeared. The software recorded the pupil and CR positions and this set of pupil-CR data was used to compute gaze positions during the trials.

Pupil size was tracked by the EyeLink software via a centroid mode, which tracks the center of the pupil image using a center-of-mass algorithm (Zhu, Moore, and Raphan 1999).

## ISA-Tab metadata file

Download metadata file

## Data Availability

All code used to import the raw data was custom-written in Matlab®, version R2014a. Code is available upon request.

## References

[1] American Psychiatric Association, A. P. A. *The Diagnostic and Statistical Manual of Mental Disorders DSM-5*. 5th edn, (American Psychiatric Publishing, 2013).

[2] Friedman LA, Rapoport JL (2015). Brain development in ADHD. Curr. Op. Neurobiol.

[3] Vaidya CJ (2012). Neurodevelopmental abnormalities in ADHD. Curr. Top. Beh. Neurosci..

[4] Frances AJ, Widiger T (2012). Psychiatric diagnosis: lessons from the DSM-IV past and cautions for the DSM-5 future. Ann. Rev. Clin. Psych.

[5] Livesley WJ (2010). Confusion and incoherence in the classification of personality disorder: Commentary on the preliminary proposals for DSM-5. Psych. Inj. Law.

[6] Insel TR (2009). Translating scientific opportunity into public health impact: a strategic plan for research on mental illness. Arch. Gen. Psychiat..

[7] Zhou X, Reynolds CR, Zhu J, Kamphaus RW, Zhang O (2018). Evidence-based assessment of ADHD diagnosis in children and adolescents. Appl. Neuropsych. Child.

[8] Wainstein G (2017). Pupil Size Tracks Attentional Performance In Attention-Deficit/Hyperactivity Disorder. Scientific Reports.

[9] Gabay S, Pertzov Y, Henik A (2011). Orienting of attention, pupil size, and the norepinephrine system. Attention, Perception & Psychophysics.

[10] Sara SJ, Bouret S (2012). Orienting and reorienting: the locus coeruleus mediates cognition through arousal. Neuron.

[11] Papesh MH, Goldinger SD (2012). Pupil-BLAH-metry: cognitive effort in speech planning reflected by pupil dilation. Attention, Perception & Psychophysics.

[12] Peysakhovich V, Causse M, Scannella S, Dehais F (2015). Frequency analysis of a task-evoked pupillary response: Luminance-independent measure of mental effort. Int. J. Psychophys.

[13] Reimer J (2014). Pupil fluctuations track fast switching of cortical states during quiet wakefulness. Neuron.

[14] Siegle GJ, Steinhauer SR, Stenger VA, Konecky R, Carter CS (2003). Use of concurrent pupil dilation assessment to inform interpretation and analysis of fMRI data. Neuroimage.

[15] Zenon A, Sidibe M, Olivier E (2014). Pupil size variations correlate with physical effort perception. Frontiers Beh. Neurosci.

[16] Rojas-Líbano, D., Wainstein, G. & Ossandón, T. Eye-tracking and Neuropsychological Dataset from ADHD-diagnosed and control participants performing a cognitive task. *figshare*, 10.6084/m9.figshare.7218725.v3 (2019). 10.1038/s41597-019-0037-2 PMC6472382 30975993

[17] Dolcos F, McCarthy G (2006). Brain systems mediating cognitive interference by emotional distraction. J. Neurosci..

